# Supplementary material for: Super-Resolution Imaging of Fas/CD95 Reorganization Induced by Membrane-Bound Fas Ligand Reveals Nanoscale Clustering Upstream of FADD Recruitment
Source: Cells. 2022 Jun 12;11(12):1908. doi: 10.3390/cells11121908 (PMC9221696; doi:10.3390/cells11121908)
Supplement: Supplementary file 1 [file cells-11-01908-s001.zip › cells-1690927-supplementary after proof.pdf]

cells MDPI

Article

Super-resolution imaging of Fas/CD95 reorganization induced by membrane-bound Fas ligand reveals nanoscale clustering upstream of FADD recruitment

Nicholas Frazzette*[1], Anthony C. Cruz*[1], Xufeng Wu[2], John A. Hammer[2] Jennifer Lippincott-Schwartz[3], Richard M. Siegel*[1,4] and Prabuddha Sengupta*[3]

Supplemental Data

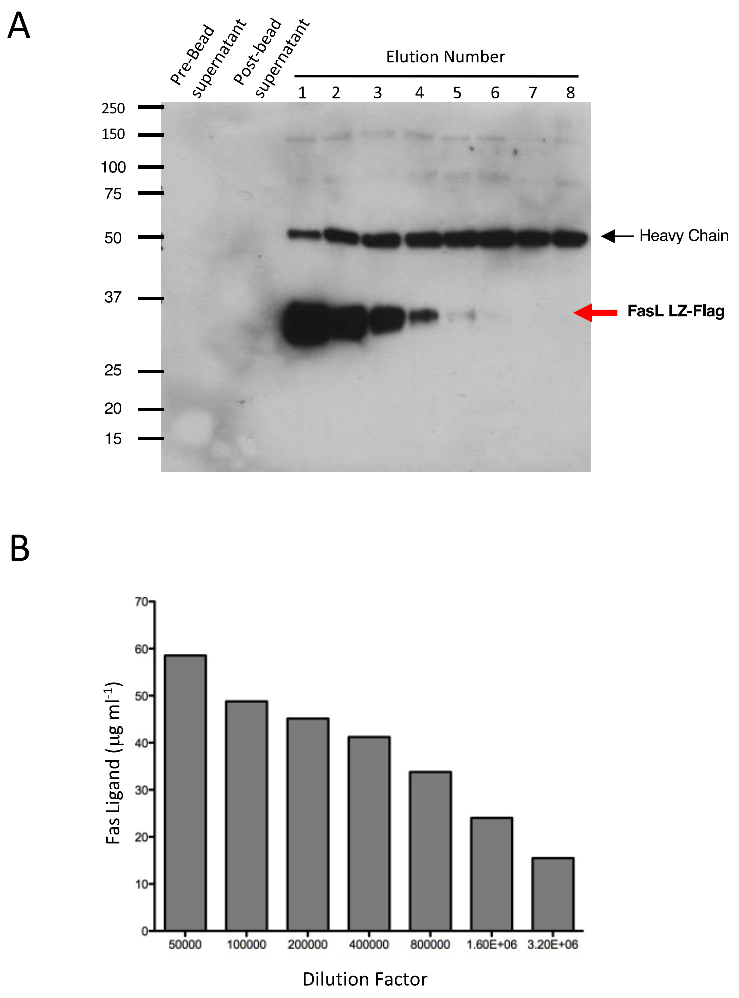

Supplemental Figure S1. Production and purification of recombinant FasL-LZ. **(A)** Western blot analysis of HEK293T cell supernatants 7d post-transfection with an expression construct for FasL-LZ tagged with a FLAG-tag. FasL-LZ was purified using anti-FLAG beads and eluted using 0.1M glycine-HCl, pH2.5. **(B)** FasL-LZ was pooled from positive elution fractions, dialyzed in PBS to remove the glycine and assayed for FasL protein levels by ELISA. An example dilution series is shown.

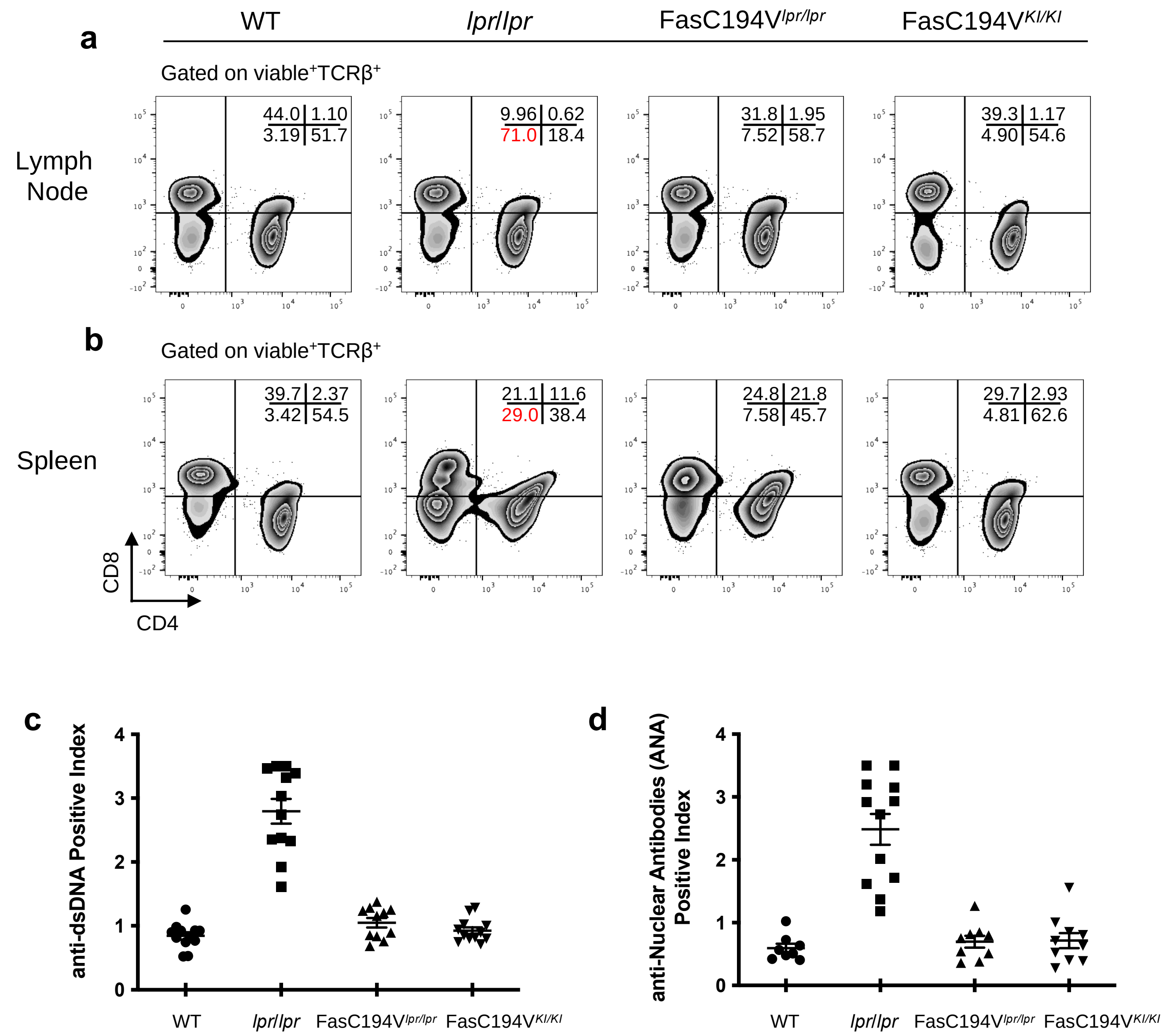

Supplemental Figure S2. Characterization of Fas C194V knock-in mice **(A, B)** Lymph nodes **(A)** or Spleen **(B)** of age-matched WT, *lpr/lpr*, FasC194V$^{lpr/lpr}$, or FasC194V$^{KI/KI}$ mice were isolated, stained for the indicated markers and analyzed by flow cytometry. Cells were gated on the viable, TCRβ$^+$ single cell population and representative of three independent experiments (N=3). **(C, D)** Serum was collected from mice of the indicated genotypes (≥ 6 months of age, N ≥ 10 mice for each genotype) and analyzed via ELISA for antibodies to double-stranded DNA (dsDNA; **C**) or antibodies to nuclear proteins (ANA; **D**). Experiment is cumulative of three independent experiments (N=3). .
